# The Role of Whole Blood Impedance Aggregometry and Its Utilisation in the Diagnosis and Prognosis of Patients with Systemic Inflammatory Response Syndrome and Sepsis in Acute Critical Illness

**DOI:** 10.1371/journal.pone.0108589

**Published:** 2014-09-30

**Authors:** Gareth R. Davies, Gavin M. Mills, Matthew Lawrence, Ceri Battle, Keith Morris, Karl Hawkins, Phylip Rhodri Williams, Simon Davidson, Dafydd Thomas, Phillip Adrian Evans

**Affiliations:** 1 NISCHR Haemostasis Biomedical Research Unit (HBRU), Morriston Hospital, Swansea, Wales, United Kingdom; 2 Institute of Life Science, College of Medicine, Swansea University, Singleton Park, Swansea, Wales, United Kingdom; 3 Intensive Therapy Unit, Abertawe Bro Morgannwg University Health Board, Swansea, Wales, United Kingdom; 4 School of Applied Science, University of Wales Institute Cardiff, Cardiff, Wales, United Kingdom; 5 College of Engineering, Swansea University, Singleton Park, Swansea, Wales, United Kingdom; 6 Department of Haematology, Royal Brompton and Harefield NHS Foundation Trust, London, United Kingdom; 7 Cardiac Intensive Care Unit, Abertawe Bro Morgannwg University Health Board, Swansea, Wales, United Kingdom; University of Leuven, Belgium

## Abstract

**Objective:**

To assess the prognostic and diagnostic value of whole blood impedance aggregometry in patients with sepsis and SIRS and to compare with whole blood parameters (platelet count, haemoglobin, haematocrit and white cell count).

**Methods:**

We performed an observational, prospective study in the acute setting. Platelet function was determined using whole blood impedance aggregometry (multiplate) on admission to the Emergency Department or Intensive Care Unit and at 6 and 24 hours post admission. Platelet count, haemoglobin, haematocrit and white cell count were also determined.

**Results:**

106 adult patients that met SIRS and sepsis criteria were included. Platelet aggregation was significantly reduced in patients with severe sepsis/septic shock when compared to SIRS/uncomplicated sepsis (ADP: 90.7±37.6 vs 61.4±40.6; p<0.001, Arachadonic Acid 99.9±48.3 vs 66.3±50.2; p = 0.001, Collagen 102.6±33.0 vs 79.1±38.8; p = 0.001; SD ± mean)). Furthermore platelet aggregation was significantly reduced in the 28 day mortality group when compared with the survival group (Arachadonic Acid 58.8±47.7 vs 91.1±50.9; p<0.05, Collagen 36.6±36.6 vs 98.0±35.1; p = 0.001; SD ± mean)). However haemoglobin, haematocrit and platelet count were more effective at distinguishing between subgroups and were equally effective indicators of prognosis. Significant positive correlations were observed between whole blood impedance aggregometry and platelet count (ADP 0.588 p<0.0001, Arachadonic Acid 0.611 p<0.0001, Collagen 0.599 p<0.0001 (Pearson correlation)).

**Conclusions:**

Reduced platelet aggregometry responses were not only significantly associated with morbidity and mortality in sepsis and SIRS patients, but also correlated with the different pathological groups. Whole blood aggregometry significantly correlated with platelet count, however, when we adjust for the different groups we investigated, the effect of platelet count appears to be non-significant.

## Introduction

Sepsis is a life threatening condition and common complication of critical illness [1]. It is characterised by a systemic inflammatory response to an ongoing infectious process and can lead to hypotension, multi organ dysfunction (MOD) and death [2]. Systemic inflammation can also have non-infectious causes such as burns, pancreatitis and ketoacidosis, and is termed systemic inflammatory response syndrome (SIRS). Prevalence of SIRS due to sterile or infectious causes is very high, affecting up to one third of all hospital patients [3] and it remains a challenge to differentiate sepsis from sterile SIRS which is vital in guiding effective treatment.

It has been shown previously that coagulation is activated across the septic range [4], [5] This activation can express itself as either a mildly increased risk of thrombosis to systemic formation of intravascular thrombi, known as disseminated intravascular coagulation (DIC). Altered coagulation contributes to the pathogenesis and outcome of sepsis. In severe sepsis microthrombi formation in the vasculature alters perfusion of blood into the organs, contributing to multiple organ dysfunction syndrome (MODS) [6]. Sepsis is also the leading cause of thrombocytopenia [7] which is related to poor outcome [8]. Studies have highlighted that the function of platelets goes beyond haemostatic regulation [9], [10] and there is increasing evidence that platelets are key mediators of inflammation and the immunological response to infection [11]. Platelet aggregation is enhanced in the presence of lipopolysaccharide (LPS) in vitro [12], which has been identified to be dependent on toll-like receptor 4 pathway [13]. This suggests increased platelet aggregation measurements might be observed in patients with sepsis.

The role of platelet aggregation is very important in inflammation. In severe sepsis, platelet aggregation has been often shown to be decreased [14] [15] [16]; however, this has yet to be investigated in the whole sepsis spectrum. Whole blood impedance aggregometry (multiplate) is a point of care test that can be used to measure platelet aggregation in response to different agonists and has been shown to be a predictor of diagnosis and prognosis in patients with severe sepsis [15]. However, it has also been suggested that whole blood impedance aggregometry is dependent on whole blood parameters (platelet count, haematocrit, haemoglobin and white cell count) [17] [18]. Of particular relevance to whole blood impedance aggregometry are platelet counts of less than 150×10^9^/L [19].

The primary aim of this study was to assess the diagnostic and prognostic accuracy of whole blood aggregometry in patients who present across the septic range and to compare these results against the whole blood parameters (platelet count, white cell count, haematocrit and haemoglobin). The secondary aim of the study was to investigate the dependence of whole blood aggregometry on the whole blood parameters.

## Materials and Methods

### Ethical Approval

Full ethical approval was given by the South West Wales Research Ethics Committee. Informed 2-stage written consent was given by patients with capacity to do so. Assent was obtained from personal or legal representation in cases where capacity to give informed consent was lacking.

### Patients

A total of 106 patients were recruited from October 2011 to November 2013 in a large teaching hospital in Wales. All patients were considered eligible as per the SIRS criteria defined in 2003 [20]. 42 healthy volunteers from a similar demographic population group and matched for gender and age were recruited as a control group. Patients with any disease that affects the coagulation profile including liver cirrhosis and renal disease were excluded. Patients that had received anticoagulant therapy were also excluded. Patient outcome was evaluated at 28 days.

Sepsis-related Organ Failure Assessment (SOFA) score [21] was determined over the first 24 hours to assess organ function. Patients were assigned to groups as follows: 1) Sterile SIRS 2) Uncomplicated sepsis 3) Severe sepsis 4) Septic shock as per the criteria defined in 2003 [20]. Assignment into groups was blinded and performed by an experienced intensive care specialist independent of the study. All platelet aggregation data was also validated independently.

### Blood Sampling

Blood was drawn at time of admission to the emergency department or intensive care unit, at 6 hours and 24 hours post admission to assess disease progression and the effect of treatment. For multiplate analysis, blood was drawn into 3mL hirudin blood tubes (Verum Diagnostica, Munich, Germany) as per manufacturers' recommendations. Testing was performed 20 minutes after the blood was drawn. A total volume of 300 µL of hirudinised blood was added to 300 µL normal saline in the multiplate test cell, which was then incubated at 37°C for 3 minutes. Blood samples were then activated using three different reagents 1) adenosine diphosphate (ADP test, Verum Diagnostica GmbH), 2) arachadonic acid test of cyclooxygenase activity (ASPI test, Verum Diagnostica GmbH) and 3) collagen (COL test, Verum Diagnostica GmbH) agonists. Aggregometry measurements were assessed via the area under the curve measurement (arbitrary units).

A 4 ml aliquot of blood was taken for full blood count (FBC) analysis to determine all the whole blood parameters. Samples were collected into plastic, full-draw dipotassium EDTA Vacuettes (Greiner Bio-One, Stonehouse, UK Ref: 454286). FBC was analysed using a Sysmex XE 2100 (Sysmex UK, Milton Keynes, UK) automated haematology analyser within 2 hrs of collection.

### Statistical Analysis

All statistical analysis was carried out on GraphPad Prism version 6 (GraphPad software, La Jolla, CA, USA). Values are given as mean and standard deviation or alternatively median and quartiles. For continuous variables T-test was used to detect significant differences between normally distributed groups and Kruskal-Wallis was used to detect differences in non-normally distributed data. Data normality was assessed using the Kolmogorov-Smirnov and Shapiro-Wilk tests using an α of 0.05. Bonferroni post hoc correction was used to assess multiple comparisons across groups. Receiver operating characteristics were determined to assess discrimination between groups. Sensitivities and specificities were determined from the optimal cut-off values, which were calculated as the point at which the Youden Index was maximised [22].

Pearson's correlation coefficient was used to investigate relationships between whole blood aggregometry and whole blood parameters. Analysis of Covariance (ANCOVA) was used to adjust for changes in platelet aggregation in the different groups investigated, with the platelet count as a covariate using the General Linear Model method.

To adjust for the effect of low platelet count on whole blood impedance aggregometry, data was reanalysed for patients with platelet counts greater than 100×10^9^/L only.

Statistical significance was defined as a p-value of less than 0.05 (two-tailed).

## Results

Platelet aggregometry and full blood count were assessed in a total of 106 patients that met SIRS criteria. This included 21 SIRS, 42 uncomplicated sepsis, 17 severe sepsis and 26 septic shock patients. A healthy group of 42 volunteers was also recruited. Clinical characteristics are presented in Table 1.

**Table 1:** Demographics of Subject Groups.

	Healthy	SIRS	Sepsis	Severe Sepsis	Septic Shock
**Number**	42	21	42	17	26
**Male (%)**	21 (50.0)	12 (57.1)	19 (45.2)	8 (47.1)	16 (61.5)
**Age (Years)**	60.1±16.5	62.8±20.4	56.6±19.6	63.9±21.4	68.6±13.0
**Primary Site of Infection**					
Respiratory Tract (%)	-	-	27 (64.3)	10 (58.8)	10 (38.5)
Urinary Tract (%)	-	-	7 (16.7)	4 (23.5)	6 (23.1)
GI Tract (%)	-	-	3 (7.1)	0 (0)	6 (23.1)
Other (%)	-	-	5 (11.9)	3 (17.6)	4 (15.4)
**Comorbidities**					
Diabetes Mellitus (%)	0 (0)	12 (57.1)	4 (9.5)	4 (23.5)	9 (34.6)
COPD (%)	0 (0)	3 (14.3)	19 (45.2)	2 (11.8)	4 (15.4)
Congestive Heart Failure (%)	0 (0)	2 (9.5)	1 (2.4)	0 (0)	2 (7.7)
Active Cancer (%)	0 (0)	0 (0)	1 (2.4)	0 (0)	3 (11.5)
**28 Day Mortality (%)**	-	2 (9.5)	1 (2.4)	0 (0)	3 (11.5)
**SOFA Score**	-	3 (2, 7)	3 (1.25, 3)	5 (4, 6)	9 (6, 12)
**Hospital LOS (days)**	-	6 (4.5, 14.5)	5 (2, 8.5)	9 (5.5, 19)	14 (2.5, 43)

COPD  =  Chronic Obstructive Pulmonary Disease; SOFA  =  Sepsis-relates Organ Failure Assessment; LOS  =  Length of Stay.

### Platelet Aggregometry for Diagnosing Sepsis Severity

**Figure 1:**
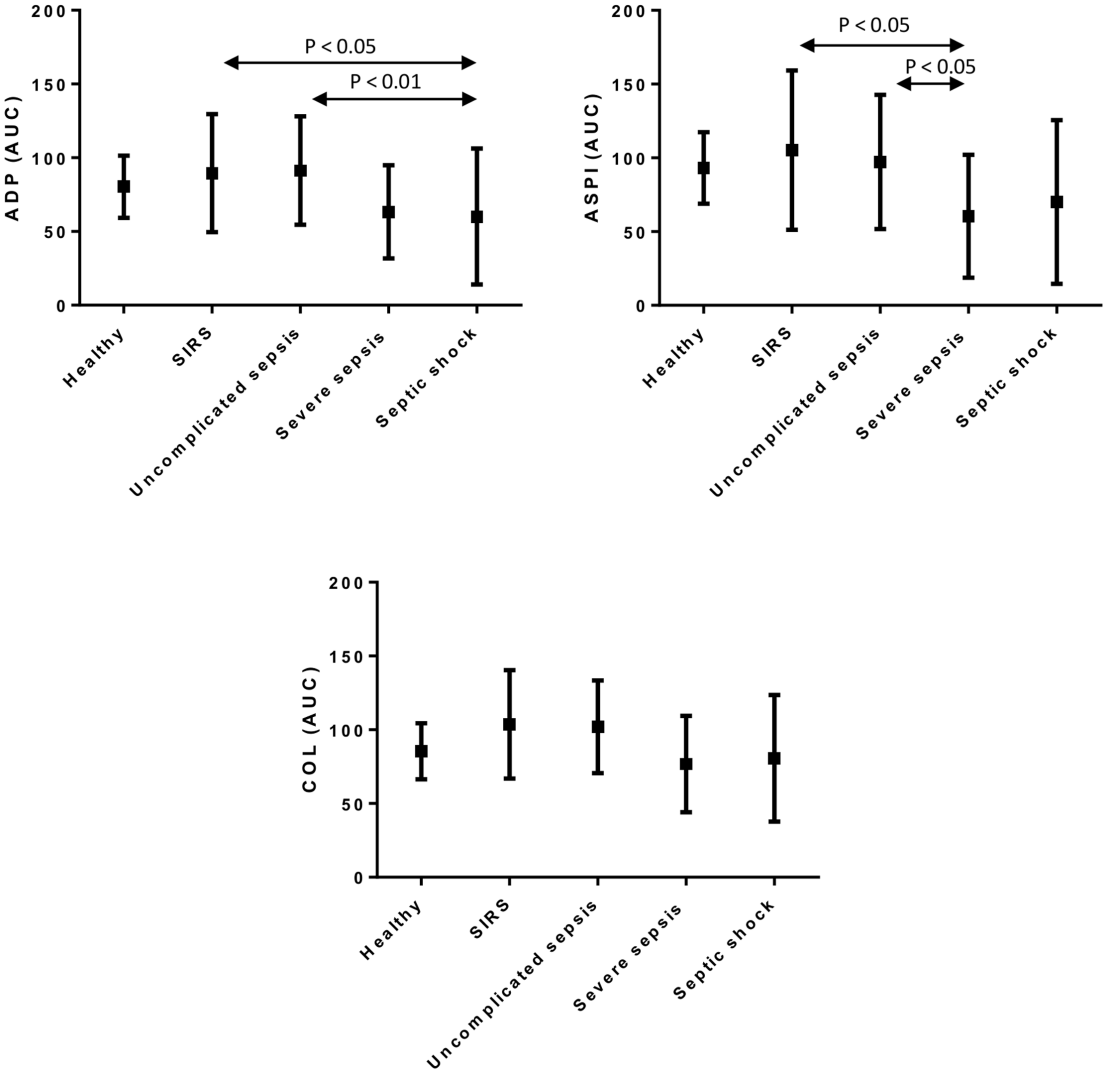
Comparison of platelet aggregometry measurements in different pathological groups and healthy control. Platelet aggregation measurements induced by ADP, ASPI and Collagen agonists are shown in the different pathological groups and healthy control. Aggregometry measurements are expressed as area under the curve (AUC, arbitrary units). Significant differences between the groups are indicated by p-values, as assessed by Bonferroni post-hoc analysis.

The results, shown in Figure 1, demonstrate reduced platelet aggregometry values in patients with severe sepsis and septic shock when compared to SIRS and uncomplicated sepsis when ADP and ASPI were used as the agonist, but this was not the case when Collagen was used as the agonist. Platelet aggregometry values in the patient groups were comparable to those of the healthy group.

**Figure 2:**
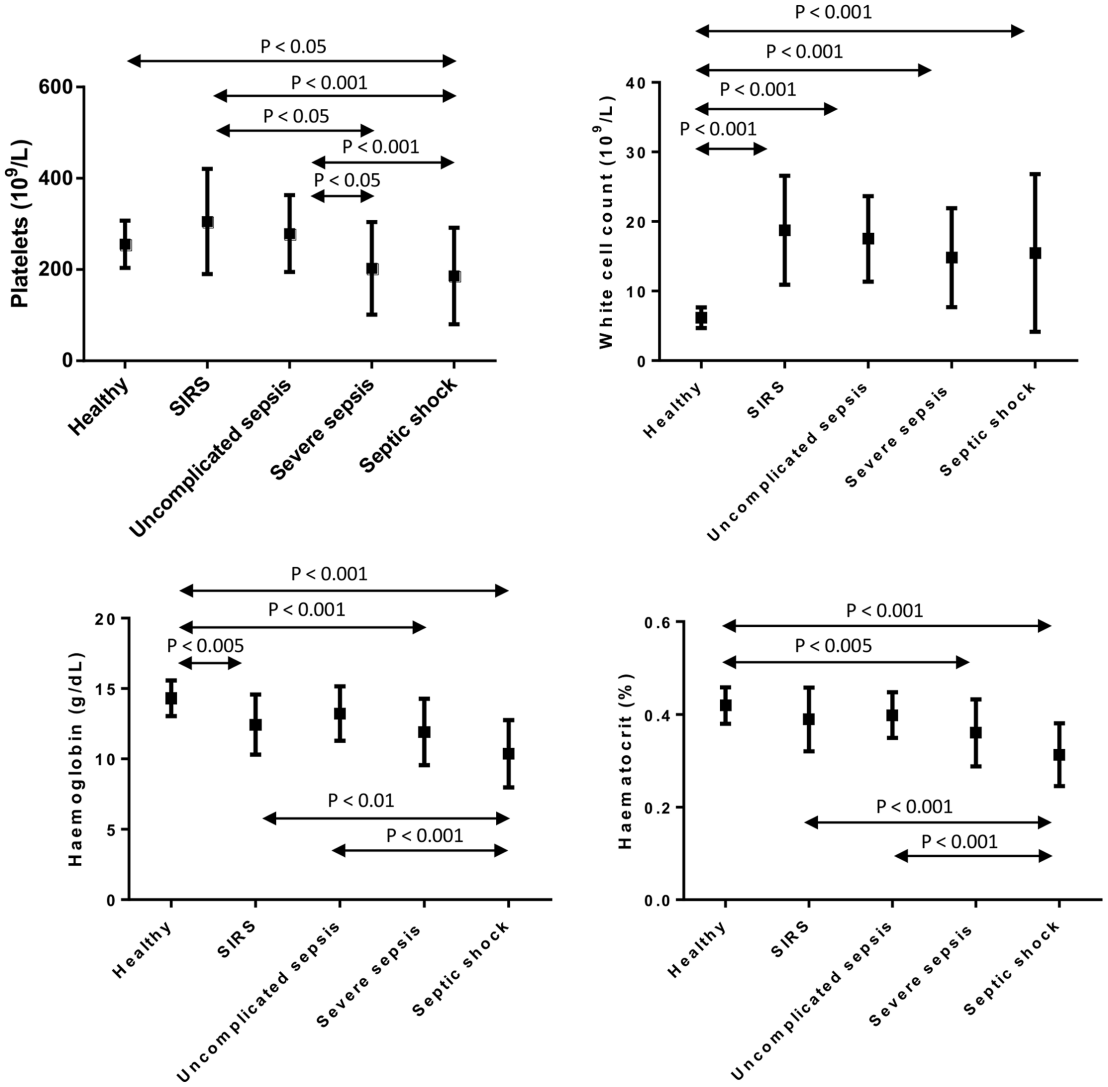
Comparison of whole blood parameters in different pathological groups and healthy control. Platelet count, white cell count, haemoglobin and haematocrit measurements are shown in different pathological groups and healthy control. Significant differences between the groups are indicated by p-values, as assessed by Bonferroni post-hoc analysis.

Results for haemoglobin, haematocrit, platelet count and white cell count are presented in Figure 2. Significant progressive reductions in haemoglobin, haematocrit and platelet count were observed across the sepsis spectrum, with increasing severity. White cell count was elevated in all patient groups when compared to the healthy group.

### Platelet Aggregometry for the Diagnosis of Severe Sepsis

To assess platelet aggregometry as a diagnostic marker of severe sepsis, patients were assigned to two groups: SIRS/uncomplicated sepsis and severe sepsis/septic shock. The results, displayed in Table 2, show significantly reduced platelet aggregation, haemoglobin, haematocrit and platelet count in patients with severe sepsis/septic shock when compared to SIRS/uncomplicated sepsis patients.

**Table 2:** Comparison of SIRS/Uncomplicated sepsis and severe sepsis/septic shock.

	Healthy (n = 42)	SIRS/Uncomplicated Sepsis (n = 62)	Severe Sepsis/Septic Shock (n = 43)	Significance value (Healthy vs SIRS/Uncomplicated)	Significance Value (SIRS/Uncomplicated vs Severe/Septic Shock
**ADP (AUC)**	80.4±21.1	90.7±37.6	61.4±40.6	NS	<0.001
**ASPI (AUC)**	93.2±24.3	99.9±48.3	66.3±50.2	NS	0.001
**COL (AUC)**	85.5±19.0	102.6±33.0	79.1±38.8	0.01	0.001
**Platelet Count (10^9^/L)**	255.6±51.9	295.0±110.9	192.5±103.2	0.03	<0.001
**White Cell Count (10^9^/L)**	6.2±1.5	17.9±6.7	15.2±9.9	<0.001	NS
**Haemoglobin (g/dL)**	14.3±1.3	13.0±2.0	11.0±2.5	<0.001	<0.001
**Haematocrit (%)**	0.42±0.04	0.39±0.06	0.33±0.07	0.01	<0.001

**Figure 3:**
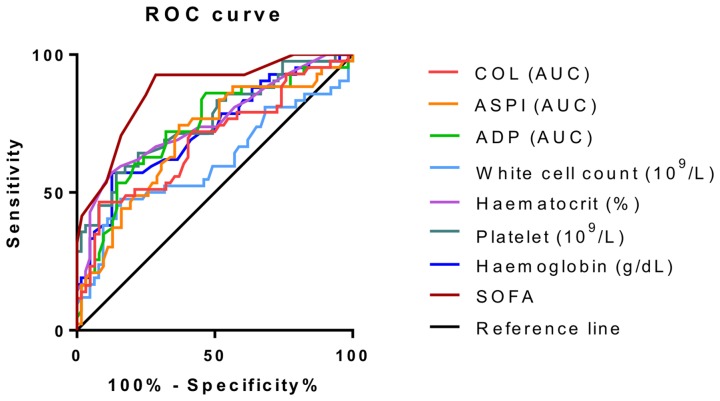
Receiver operating characteristics for discrimination between SIRS/Uncomplicated Sepsis and Severe Sepsis/Septic Shock groups. Receiver operating characteristics are shown for platelet aggregometry measurements, whole blood parameters and sepsis-related organ failure assessment (SOFA) score as a discriminator between patients with and without organ dysfunction.

To further assess discrimination between SIRS/uncomplicated sepsis and severe sepsis/septic shock groups, receiver operating characteristics were analysed (Figure 3). The corresponding area under the curve and significance are shown in Table 3. All parameters except for white cell count showed significant discrimination between groups as defined by p-values of less than 0.05. This indicates that whole blood impedance aggregometry is able to distinguish between those with SIRS/uncomplicated sepsis and severe sepsis/septic shock, particularly when ADP is used as the agonist. The most significant discriminatory power, apart from SOFA score, was observed with ADP aggregometry, haematocrit and platelet count.

**Table 3:** Receiver Operating Characteristics for the Diagnosis of Severe Sepsis.

Test Result Variable(s)	Area	Std. Error	Asymptomatic Sig.	Asymptomatic 95% Confidence Interval (Lower Bound)	Asymptomatic 95% Confidence Interval (Upper Bound)
**ADP (AUC)**	0.740	0.051	<0.001	0.640	0.840
**ASPI (AUC)**	0.697	0.054	0.001	0.592	0.802
**COL (AUC)**	0.689	0.055	0.001	0.582	0.796
**Platelet Count (10^9^/L)**	0.759	0.049	<0.001	0.664	0.855
**White Cell Count (10^9^/L)**	0.608	0.060	0.061	0.491	0.726
**Haemoglobin (g/L)**	0.722	0.052	<0.001	0.620	0.823
**Haematocrit (%)**	0.746	0.051	<0.001	0.646	0.846
**SOFA**	0.870	0.036	<0.001	0.799	0.942

SOFA  =  Sepsis-related Organ Failure Assessment score.

Optimal cut-off points were determined as described in the methods section. Sensitivity, specificity and the optimal cut-off for each parameter are displayed in Table 4.

**Table 4:** Sensitivity and Specificity for the Diagnosis of Severe Sepsis.

Cutoff	Sensitivity (%)	Specificity (%)
**ADP <75.5**	75.6	71.9
**ASPI <85.5**	73.2	64.9
**COL <62.5**	48.8	93
**Platelet Count <211.5**	61	80.7
**White Cell Count <12.35**	48.8	82.5
**Haemoglobin <11.35**	58.5	86
**Haematocrit <0.322**	53.7	91.2
**SOFA>3.5**	92.7	71.9

Sensitivity: Percentage of severe sepsis patients with a positive test.

Specificity: Percentage of SIRS/uncomplicated sepsis patients with a negative test.

SOFA  =  Sepsis-related Organ Failure Assessment score.

### Platelet Aggregometry for Determining Prognosis in Sepsis Patients

**Figure 4:**
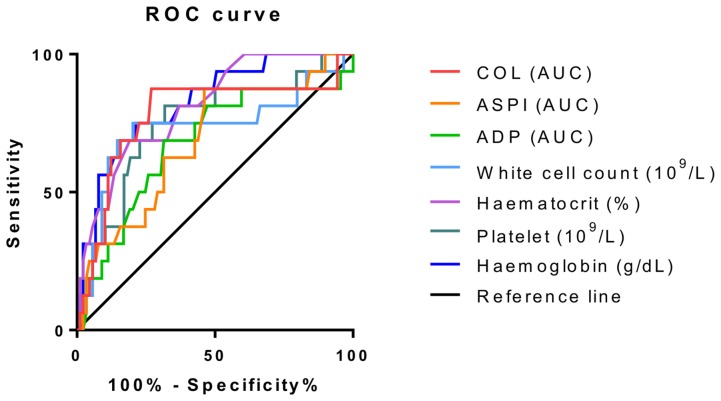
Receiver operating characteristics for discrimination between survivors and non-survivors at 28 days. Receiver operating characteristics are shown for platelet aggregometry measurements and whole blood parameters for the discrimination between 28-day mortality and survival.

All patients were followed up for 28-day mortality. Characteristics of 28 day survival and mortality groups are shown in Table 5. Platelet aggregometry was significantly reduced in the 28-day mortality group using ASPI and COL agonists; however, with ADP activation this did not appear to be the case. Haemoglobin, platelet count and white cell count were all significantly reduced in the 28-day mortality group. Receiver operating characteristic curves showing discrimination between survivors and non-survivors are shown in Figure 4. The AUC, significance and confidence intervals are shown in Table 6. All measured parameters showed significant discrimination between survivors and non-survivors (Asymptomatic Sig <0.05). This indicates reduced whole blood parameters and impaired platelet function are strongly related to poor outcome in sepsis patients.

**Table 5:** Characteristics of Survivors and Non-Survivors in Relation to Platelet Aggregation and Whole Blood Parameters.

	28 Day Survival (n = 89)	28 Day Mortality (n = 16)	Significance value
**ADP (AUC)**	81.6±39.2	62.9±49.8	0.097
**ASPI (AUC)**	91.1±50.9	58.8±47.7	0.02
**COL (AUC)**	98.0±35.1	64.8±36.6	0.001
**Platelet Count (10^9^/L)**	268.3±117.7	174.4±91.9	0.003
**White Cell Count (10^9^/L)**	17.8±7.6	11.3±9.2	0.003
**Haemoglobin (g/dL)**	12.6±2.2	9.7±2.1	<0.001
**Haematocrit (%)**	0.38±0.06	0.30±0.06	<0.001

Comparisons were made using two-sample t-test.

**Table 6:** Receiver Operating Characteristics for Survivors and Non-Survivors for Platelet Aggregation and Whole Blood Parameters.

Test Result Variable(s)	Area	Std. Error	Asymptomatic Sig.	Asymptomatic 95% Confidence Interval (Lower Bound)	Asymptomatic 95% Confidence nterval (Upper Bound)
**ADP (AUC)**	.675	.079	0.027	.521	.829
**ASPI (AUC)**	.686	.072	0.018	.546	.827
**COL (AUC)**	.777	.075	<0.001	.631	.924
**Platelet Count (10^9^/L)**	.750	.068	0.001	.616	.884
**White Cell Count (10^9^/L)**	.731	.083	0.003	.568	.895
**Haemoglobin (g/L)**	.822	.055	<0.001	.714	.930
**Haematocrit (%)**	.811	.053	<0.001	.707	.914

### Effect of Treatment on Platelet Aggregation

**Figure 5:**
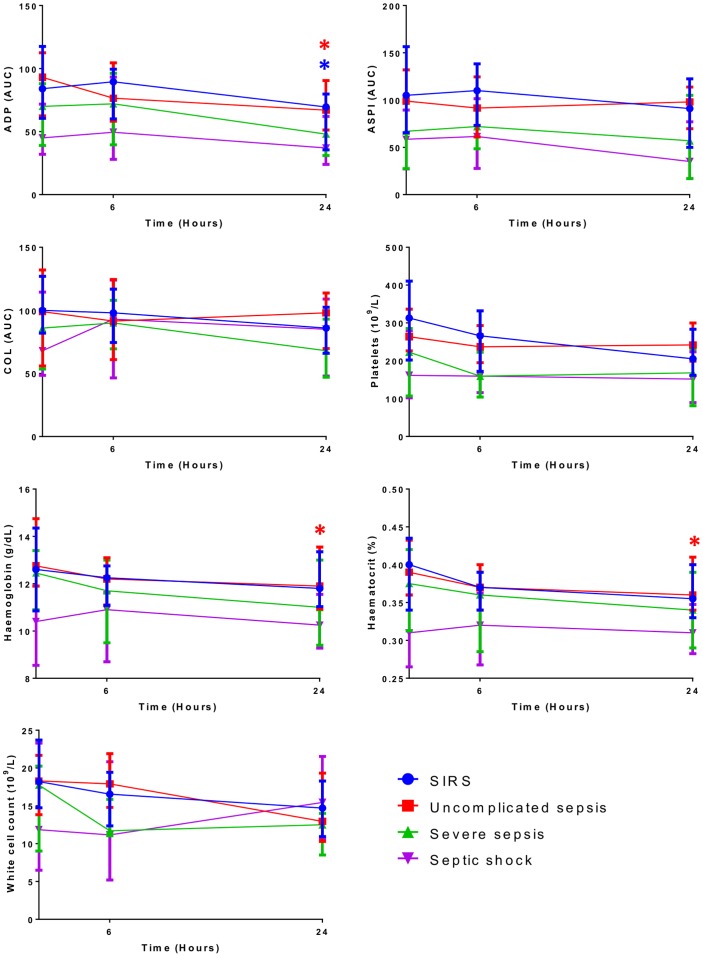
Effect of treatment and disease progression on platelet aggregation and whole blood parameters. Platelet aggregation and whole blood parameters were measured at baseline, 6 hours and 24 hours are shown. *Significant result (P<0.05), two-sample t-test.

To assess the effect of treatment and disease progression on platelet aggregation, measurements were repeated at 6 and 24 hours post admission. The results, shown in Figure 5, demonstrate a significant reduction in ADP induced aggregation over 24 hours in the SIRS and uncomplicated sepsis groups. Haemoglobin and haematocrit were also significantly reduced over 24 hours in uncomplicated sepsis. No other significant changes were observed in any of the groups over 24 hours. Reduced platelet aggregation and whole blood parameters were observed in the more severe groups over the 24 hours; however, this was not significant.

### Dependence of Platelet Aggregometry on Whole Blood Parameters

Correlation analysis was used to assess the dependence of platelet aggregometry on platelet count, haemoglobin and leukocyte count. Platelet aggregation correlated significantly with all blood count parameters (Table 7). Using correlation analysis, R2 values were 0.363, 0.420 and 0.379 (p<0.001) for ADP, ASPI and Collagen agonists respectively.

**Table 7:** Pearson's Correlations of Whole Blood Aggregometry versus Whole Blood Parameters.

Agonist		Platelet Count (10^9^/L)	White Cell Count (10^9^/L)	Haemoglobin (g/dL)	Haematocrit (%)
**ADP (AUC)**	Pearson Correlation	0.598	0.254	0.228	0.265
	Sig (2-tailed)	<0.001	0.009	0.020	0.007
**ASPI (AUC)**	Pearson Correlation	0.635	0.362	0.252	0.307
	Sig (2-tailed)	<0.001	<0.001	0.010	0.001
**Collagen (AUC)**	Pearson Correlation	0.607	0.476	0.189	0.247
	Sig (2-tailed)	<0.001	<0.001	0.055	0.012

Analysis of the effect of platelet count on platelet aggregation using all three aggregating agents and using Analysis of Covariance (General Linear Model) would suggest that the platelet count significantly affects platelet aggregation, however, the change in platelet aggregation for ADP, collagen and aspirin is significantly different in the sepsis and non-sepsis groups even when we adjust for the platelet count (P<0.005, ANCOVA). Hence, this analysis would support the view that sepsis has an effect on both the platelet count together with platelet function.

### Adjusting for Low Platelet Count

To correct for the potential effects low platelet count had on Multiplate readings, data was reanalysed for patients with a platelet count greater than 100×10^9^/L (n = 97). Platelet aggregometry using all three agonists (ADP, ASPI and collagen) remained significant at distinguishing between SIRS/uncomplicated sepsis and severe sepsis/septic shock (ADP 68.8±39.9 vs 90.7±37.6, p<0.05; ASPI 76.4±50.7 vs 99.9±48.3, p<0.05; COL 85.7±37.8 vs 102.6±33.0, p<0.05). All measured parameters except for ADP aggregometry remained significantly lower in the 28-day mortality group (p<0.05).

## Discussion

This, to our knowledge, is the first study to assess whole blood aggregometry across the entire pathological spectrum of SIRS and sepsis. The data demonstrates that a reduction in aggregometry measurements, haemoglobin and platelet count are associated with increasing severity of sepsis. Several authors have reported and reviewed that platelet function as well as platelet count is significantly modulated during sepsis-in particular severe sepsis [4], [15], [23]. Our data supports these findings in that our analysis demonstrated both a significant reduction and a significant modulation of platelet aggregation mediated through all three agonists used herein. Platelets display a number of properties besides repairing damaged vascular endothelium and preventing bleeding. It is known that platelets act to induce pro-inflammatory events [24] and can engage infectious pathogens [25]. The sophisticated interplay of platelets with bacteria may culminate in sepsis that is characterized by significant progressive reductions in platelet count and platelet function with increasing severity of the sepsis. This study supports the view that significant changes in platelet count occur during sepsis. Although there was a reduction in platelet count with progression of sepsis, our findings showed that there was also a marked decrease in platelet activity.

As a marker of prognosis, haemoglobin was found to be a highly significant predictor of 28-day mortality, which has been described previously [26]. Low platelet count, white cell count and platelet aggregometry by ASPI and collagen activation were also significantly associated with mortality, but this was not the case when ADP was used as the agonist. The significant association between ADP aggregometry and severity but not mortality is interesting and could provide insight into the progression of some of the pathogenic mechanisms of sepsis. Previous studies have suggested that anti-platelet therapy could be beneficial in critically ill patients by reducing microvascular thrombi formation and hence improving perfusion [27], [28]. It is possible that platelet responses to ADP could be blunted if platelets have been exposed to ADP in vivo, as previously it has been shown that platelets exposed to ADP become desensitised, and unresponsive to restimulation [29]. This is a cause for concern in patients in which there is a degree of haemolysis as ADP released from the red cells could cause platelet refractoriness to further agonist exposure. This could be an additional mechanism for the reduced ADP responses observed in the severe sepsis and septic shock groups.

Reduced platelet aggregometry measurements were observed in patients with severe sepsis and septic shock when compared to SIRS and uncomplicated sepsis. Previously it has been hypothesised that reduced platelet aggregation might be observed in severe sepsis and septic shock as consumption of platelets with increased activation occurs due to the hypercoagulable state and endothelial dysfunction [30]. This suggests that platelet aggregometry could be a biomarker of endothelial function and the level of ongoing DIC. The findings in this study support previous studies that have observed reduced platelet aggregometry in patients with severe sepsis [15], [16]. Furthermore, this study demonstrates that platelet aggregometry measurements using collagen as the agonist were significantly increased in patients with uncomplicated sepsis/sirs compared to healthy volunteers. However, this was not the case when using ADP or ASPI agonists. The data presented in this study suggests that platelet count and haemoglobin are equally as effective as whole blood impedance aggregometry as diagnostic and prognostic tools in patients that meet SIRS and sepsis criteria. The strong relationship between haemoglobin and sepsis severity and outcome is an interesting one. It is clear from the literature that red blood cell derangement is common occurrence in severe sepsis and this contributes to impairment of the microcirculation leading to eryptosis and reduced circulating haemoglobin [31], [32]. Consequently haemoglobin measurements could be providing information on micro perfusion and hence organ dysfunction. Recent studies has also highlighted the impact low haemoglobin level have on blood CO2 binding capacity [33]. This suggests anaemia could contribute to acidosis, and enhance coagulopathy in sepsis.

We observed that different agonists yielded different operating characteristics when assessing diagnosis and prognosis. This can be explained as each of these agonists causes activation of platelets via a different route e.g. ADP through the P2Y receptors, ASPI through COX-1 cyclooxygenase pathway and Collagen through glycoprotein VI and therefore display different strengths in their ability to activate platelets, some being more effective activators than others. In SIRS/sepsis patients, it could be the case that this variability is heightened.

When assessing the effect of platelet count on platelet aggregation, significant correlations were observed. Furthermore, when excluding subjects with a low platelet count from the analysis, platelet aggregation remained significantly reduced with relation to severity and poor outcome. Although platelet aggregation values are affected by platelet count, variability exists in both platelet count and platelet function in the healthy range and it is also possible to achieve normal aggregometry readings at low platelet counts [18]. Therefore, taking into account the limitations of the assay, there remains a trend to significantly reduced platelet function with increasing severity of disease in this study population.

### Limitations

This large pilot observational study has a number of limitations. The inherent problem of this and other reported studies is the heterogeneity of the disease, treatment and comorbidities. It is also difficult to take into account differences in medication and treatment between groups, and to assess this fully, a much larger number of patients would be required. In order to overcome some of these limitations we implemented tight inclusion/exclusion criteria and excluded patients that were receiving anticoagulant therapy at baseline. Sample size could be viewed as a limitation in this study; however, the sample size was powerful enough for us to detect several significant differences between groups. Further larger prospective studies are required to build on the findings of this study.

## Conclusions

This study supports the view that significant changes in platelet function occur across the sepsis spectrum, possibly due to different pathophysiological mechanisms. There remains a trend towards worsening platelet function with increasing severity in patients with SIRS and sepsis that is independent of platelet count.

Furthermore, our study demonstrates that haemoglobin and platelet count have greater diagnostic and prognostic value than whole blood impedance aggregometry measurements in patients with SIRS and sepsis. This research further highlights the importance of red blood cells and platelet count in patients with SIRS and sepsis and the potential benefits therapeutic intervention addressing red blood cell rheology and function could have. Further larger studies are now required to explore and determine how the mechanistic role of platelet activity and change across the sepsis spectrum is related to the disease process and outcome.
